# Suppression of FOXP3 expression by the AP-1 family transcription factor BATF3 requires partnering with IRF4

**DOI:** 10.3389/fimmu.2022.966364

**Published:** 2022-08-25

**Authors:** Preston R. Arnold, Mou Wen, Lei Zhang, Yuanlin Ying, Xiang Xiao, Xiufeng Chu, Guangchuan Wang, Xiaolong Zhang, Zhuyun Mao, Aijun Zhang, Dale J. Hamilton, Wenhao Chen, Xian C. Li

**Affiliations:** ^1^ College of Medicine, Texas A&M Health, Bryan, TX, United States; ^2^ Immunobiology and Transplant Science Center and Department of Surgery, Houston Methodist Hospital, Texas Medical Center, Houston, TX, United States; ^3^ Department of Medicine, Houston Methodist, Weill Cornell Medicine Affiliate, Houston, TX, United States; ^4^ Center for Bioenergetics, Houston Methodist Hospital and Research Institute, Texas Medical Center, Houston, TX, United States; ^5^ Weill Cornell Medical College of Cornell University, New York, NY, United States; ^6^ Department of Surgery, Weill Cornell Medical College of Cornell University, New York, NY, United States

**Keywords:** Foxp3, BATF family, IRF4, super enhancer, regulatory T (Treg) cell, glycolytic reprogramming, cell fate decision, TCR signaling

## Abstract

FOXP3 is the lineage-defining transcription factor for Tregs, a cell type critical to immune tolerance, but the mechanisms that control FOXP3 expression in Tregs remain incompletely defined, particularly as it relates to signals downstream of TCR and CD28 signaling. Herein, we studied the role of IRF4 and BATF3, two transcription factors upregulated upon T cell activation, to the conversion of conventional CD4+ T cells to FOXP3+ T cells (iTregs) *in vitro*. We found that IRF4 must partner with BATF3 to bind to a regulatory region in the *Foxp3* locus where they cooperatively repress FOXP3 expression and iTreg induction. In addition, we found that interactions of these transcription factors are necessary for glycolytic reprogramming of activated T cells that is antagonistic to FOXP3 expression and stability. As a result, *Irf4* KO iTregs show increased demethylation of the critical CNS2 region in the *Foxp3* locus. Together, our findings provide important insights how BATF3 and IRF4 interactions integrate activating signals to control CD4+ cell fate decisions and govern *Foxp3* expression.

## Introduction

CD4+ regulatory T cells (Tregs) are essential to the establishment and maintenance of peripheral tolerance and can be produced in the thymus from developing T cells (thymic or natural Tregs (tTreg)), in the periphery from naïve T cells (peripheral Tregs (pTregs)), or *in vitro* from *ex vivo* naïve T cells (induced Tregs (iTregs)). Tregs are defined primarily by the expression of the master transcription factor FOXP3. Thus, understanding the mechanisms that control FOXP3 expression is critical to understanding the development of Tregs and the maintenance of peripheral tolerance.

Skewing of recently activated CD4+ T cells to a Treg fate is dependent on the integration of signals from the T cell receptor (TCR), costimulatory molecules, and cytokines. The roles of the cytokines IL-2 and TGF-β are well established as necessary cytokines to FOXP3 expression in iTregs and pTregs. Depending on the timing and signal strength, however, costimulatory signaling from CD28 and OX40 can be inhibitory to Treg differentiation (1, 2). The role of TCR signaling in FOXP3 expression is well clearly defined with conflicting reports regarding the positive or negative impact on FOXP3 expression outside the thymus. This may be the result of the intimacy of CD28 and TCR signaling, as it has been shown that strong TCR signaling in the absence of CD28 can promote Treg generation from naïve CD4+ T cells (3). In its presence, however, strong TCR signaling is antagonistic to FOXP3 expression (4). In addition to these signals, FOXP3 expression and the Treg phenotype are modulated by the metabolic profile, with increased glycolysis and mTOR activation in response to TCR and CD28 signaling inhibitory to FOXP3 expression (5).

At the gene level, the *Foxp3* locus in both mice and humans consists of at least 4 key enhancers, termed conserved non-coding sequences (CNS) 0-3 (6, 7). CNS0 binds STAT5 in response to IL-2 (8). Together with CNS3, it drives the 3D architecture and activation of the *Foxp3* gene (9). CNS1 lies within the first intron and is not necessary for tTregs but contains a TGF-β response elements that binds SMAD3 and is necessary for pTregs and iTregs (6). CNS2 also lies within the first intron and is bound by FOXP3 itself in a demethylation dependent manner, allowing continued stability and expression of FOXP3 (6). TET enzymes have been shown to be essential for demethylation of the CNS2 region and the resultant FOXP3 stability (10, 11). Together, these enhancers span a large region of open chromatin with an H3K27ac signature that expands almost 15 kb upstream of the *Foxp3* locus and has been described as a super enhancer (7). While the roles of the transcription factors SMAD3 and STAT5 in controlling the *Foxp3* locus are well studied, the role of transcription factors downstream of TCR and CD28 signaling is less clear. Various binding sites for AP-1, NFAT, and NFκB transcription factors have been identified across the *Foxp3* promoter and enhancers and promote FOXP3 expression (12–14). However, how signals from the TCR and CD28 might limit FOXP3 expression remains poorly understood.

Interferon regulatory factor 4 (IRF4) is a transcription factor that is upregulated in relation to the strength of TCR signaling, making it a rheostat to translate the strength of TCR signaling into transcriptional programs (15). IRF4 is critical to full T cell activation, metabolic upregulation, and function but binds DNA weakly on its own and depends on interactions with other transcription factors, such as the Basic Leucine Zipper ATF-Like Transcription Factor (BATF), to fully carry out its transcriptional program (15–20). BATF and BATF3 are members of the AP-1 family of transcription factors that form dimers with JUN family proteins and are pioneer transcription factors in T cells (21, 22). BATF is expressed upon T cell activation, particularly CD28 signaling, and is promoted by OX40, IL-12 and IL-6 signaling while being inhibited by TGF-β signaling (2, 19, 21, 23, 24). BATF3 is similarly upregulated in T cells upon activation, and BATF and BATF3 are largely considered redundant as they can compensate for each other’s function (2, 25–28). They are critical to fully activated and functioning T cells, prevention of exhaustion, Th17 lineage differentiation, and can also impact T cell metabolic reprograming (17, 20, 23, 29–31). IRF4 and BATF are necessary for the effector functions of Tregs and have been shown to cooperate to translate the strength of TCR signaling into transcriptional programs (24, 32, 33). But how they contribute to FOXP3 expression is not fully known.

We and others have previously identified BATF3 as a potent inhibitor of FOXP3 (2, 28), but the molecular mechanisms involved remain unclear. Here we show that BATF3 and IRF4 interactions are critical to FOXP3 inhibition and IRF4 binding within the *Foxp3* super enhancer in iTregs. BATF3 and IRF4 interactions additionally control FOXP3 expression and stability by driving glycolytic remodeling of T cell metabolism upon activation. Thus, Batf3 and IRF4 represent an important axis for translating TCR signaling into CD4+ T cell fate decisions and Treg biology.

## Materials and methods

### Mouse strains

All mice used were from the C57BL/6 genetic background and were kept in the Houston Methodist Research Institute’s Comparative Medicine facilities according to IACUC protocols. *Foxp3gfp* mice used have been previously described and contain an IRES between *Foxp3* and *gfp* allowing for easy identification of FOXP3+ cells (34). *Batf3* transgenic mice (*Batf3* Tg) were kindly provided by Dr. E.J. Taparowsky and were generated similarly to described *Batf* Tg mice (35) and contain a myc-tagged *Batf3* behind the CD2 promoter for transgenic expression in T cells. They were maintained as heterozygous transgenic mice by breeding with a WT partner and then PCR genotyping for presence of the transgene in offspring. *Irf4* ^-/-^ (*Irf4* KO) were also previously generated as described (36). *Irf4* KO and *Batf3* Tg mice were crossed with WT reporter mice to generate *Foxp3gfp* strains similar to the WT mice. *Irf4* KO and *Batf3* Tg mice were crossed with each other to produce *Irf4* KO *Batf3* Tg mice which also contained the *Foxp3gfp* reporter. When possible, same-sex WT and *Irf4* KO littermates were used as controls in experiments involving *Batf3* Tg and *Irf4* KO-*Batf3* Tg mice. *Batf* ^-/-^ (*Batf* KO), and *Batf3* ^-/-^ (*Batf3* KO) mice were purchased from Jackson labs. *Batf* KO and *Batf3* KO were crossed to create *Batf*/*Batf3* double knockout (DKO) mice.

### Treg differentiation

Spleens and lymph nodes were taken from 6–12-week-old mice sacrificed according to IACUC protocol. Naïve CD4+ T cells were isolated as CD25-CD4+ using magnet-activated cell sorting (MACS). Cells were activated on 96 well flat-bottom CD3-coated plates with soluble CD28 at 1ug/mL. Cells were plated at a density of 0.1-0.15 million cells per well. T_0_ media consisted of RPMI media supplemented with 10% FBS, 1% P/S, 1% HEPES buffer, and 0.1% β-mercaptoethanol (0.55 µM final concentration). For iTreg media, the cytokines IL-2 and TGF-β were added to final concentrations of 10 ng/mL and 3 ng/mL, respectively. For 2-HG experiments, cells were cultured in iTreg media with octyl-2-hydroxyglutarate dissolved in DMSO or with an equivalent volume of DMSO. 2-HG was added to a final concentration of 0.5 mM.

### Retroviral transductions

*Batf3, Batf*, and *Irf4*, all with N-terminal *flag*, were cloned into the pMYs-IRES-GFP retroviral vector. The empty vector was used as a GFP control. Mutations were cloned into the *Batf3* vector using the GeneArt Site-Directed Mutagenesis PLUS kit and primers listed in **Supplemental Table 1**, according to manufacturer protocol. Successful mutagenesis was confirmed by Sanger sequencing. PE cells were transfected using Lipofectamine 3000 according to manufacturer protocol. Viral media was collected at 24, 48, and 72 hrs and stored at -80°C. Naïve CD4+ T cells were activated overnight in cytokine-free (T_0_) media. Polybrene was added to the thawed viral media at 1:1000 ratio. T_0_ media was removed from the cells and replaced with 200 μL of the viral media. Cells were spun at 900 g for 90 min and then rested at 32°C for 4 hrs. The viral media was then replaced with iTreg media. All transductions were performed in non *Foxp3gfp* reporter cells to allow confirmation of viral transduction through GFP expression.

### Immunoblots

CD4+ cells were cultured in iTreg or T_0_ media for 48 hrs, collected, washed, and nuclear and cytoplasmic proteins collected using Thermo Scientific NE-PER Nuclear and Cytoplasmic Extraction Kit or RIPA buffers according to manufacturer protocol. Extracted proteins were diluted in Thermo Scientific 5X Lane Reducing Sample Buffer and run out on Bio-Rad Mini-PROTEAN TGX Gels and transferred to PVDF membrane using the semi-dry Trans-Blot Turbo transfer system. Membranes were blocked with 5% milk or BSA and incubated overnight with antibody diluted 1:1000 in 5% milk or BSA at 4°C. Membranes were washed and incubated with anti-rabbit or anti-mouse antibodies conjugated to HRP and then captured using the KwikQuant system. Cytoplasmic/nuclear fractionation was confirmed using β-actin, HDAC1, and H3 proteins. Antibodies used are listed in **Supplemental Table 2**.

### Co-immunoprecipitation

CD4+ cells were transduced with *Batf3* WT, *Batf3* Mut, or empty vector control and incubated in iTreg inducing conditions for 48 hrs. Cells were washed and lysed in IP lysis buffer according to manufacturer protocol. An aliquot was set aside as whole cell lysate, and the remaining lysate was incubated overnight with anti-flag antibody agarose-conjugated beads. The lysate was removed, and beads were washed 3x with PBS before resuspending the beads. Beads were resuspended in Thermo Scientific 5X Lane Reducing Sample Buffer, briefly heated, and then run on immunoblot according to previous protocol.

### Electrophoretic mobility shift assay (EMSA)

293 T cells were transduced with pMYs-IRES-GFP vectors containing flag-*Batf3* WT, flag-*Batf3* Mut, *Junb*, *Irf4*, or empty vector. Nuclear proteins were collected using Thermo Scientific NE-PER Nuclear and Cytoplasmic Extraction Kit according to manufacturer protocol. Total protein levels were measured using the Bio-Rad Protein Assay according to manufacturer protocol. EMSA was completed with the Thermo Scientific Gel Shift Assay according to manufacturer protocol. Total nuclear protein was balanced in each assay using nuclear protein from GFP-transduced cells. All assays were incubated with 1 μL anti-IRF4 antibody diluted 1:10 in PBS to demonstrate super-shift at IRF4 binding. Biotinylated DNA with the AICE sequence from the CTLA4 gene was used to determine BATF3 and IRF4 binding (**Supplemental Table 1**).

### Flow cytometry

Cells were washed and stained for surface markers and cell viability. Cell were then fixed with eBioscience FoxP3/Transcription Factor Staining Buffer Set or BD Phosflow according to manufacturer protocol and stained for internal markers. Proliferating assays were run by staining cells with CellTrace Violet before culture. Antibodies used are listed in **Supplemental Table 2**.

### Chromatin immunoprecipitation

For ChIP-qPCR, WT, *Batf3* Tg, *Irf4* KO, and *Irf4* KO-*Batf3* Tg cells were cultured for 48 hrs under iTreg conditions. At 48 hrs, cells were collected, washed, checked to ensure cell viability >85% with trypan blue, and then flash frozen until further processing. Cells were processed using the Active Motif ChIP-IT PBMC kit according to manufacturer instructions. Cells were sonicated on QSonica800R3 at 90% amplitude for 1 hr total sonication time. Fragmentation was checked on 1.5% agarose gel. ChIP was conducted using antibodies in **Supplemental Table 2**. qPCR was run with SYBR green according to manufacturer protocol. Primers are listed in **Supplemental Table 1**. For ChiP-seq DKO cells were transduced with flag-*Batf3* WT, flag-*Batf3* Mut, or GFP vectors, cultured for 24 hrs in iTreg conditions, and then FACS sorted for viral positive cells by taking GFP positive cells. After sorting, cells were washed and flash frozen, and sent to Active Motif for IRF4 ChIP-seq.

### Glycolytic rate assay

Naïve WT, DKO, and *Irf4* KO CD4+ T cells were activated and cultured for 48 hrs in T_0_ conditions. Cells were collected, washed, counted, and plated on Agilent Seahorse XF96 PDL-coated microplates according to manufacturer’s instructions at a density of 0.1 million cells per well. Assay media consisted of Agilent RPMI XF media with glucose, pyruvate, and glutamate added to final concentrations of 2.5 µM, 1 µM, and 2 µM, respectively. Glycolytic Rate Assay Kit was run on Seahorse XFe 96 according to manufacturer protocol. Each sample consisted of 4-6 technical replicates per run across three independent experiments. The averages of each independent experiment were then averaged together for statistical analysis and figure generation.

### Bisulfite sequencing

Bisulfite sequencing was completed as previously described (10).

### iTreg stability assay

Naïve CD4+ T cells were differentiated in iTreg conditions on flat-bottom CD3 coated plates with soluble CD28. At 72 hrs, % FOXP3+ was measured by flow cytometry. Cells were collected, washed, counted, and then recultured in T_0_ conditions with CD3-negative mitomycin-treated splenocytes as antigen presenting cells (APCs) to provide CD28 costimulation and soluble anti-CD3 antibody at 1.5 µg/mL concentration for TCR stimulation. Cells were cocultured at a 1:1 ratio of APC:iTreg in round-bottom plates. After 72 hrs, cells were again collected, washed, and % FOXP3+ of live CD4+ T cells was determined. % initial FOXP3 expression was calculated as the FOXP3+ % after coculturing in T_0_ conditions with APCs divided by the FOXP3+ % before coculture.

### Statistical analysis

All statistics were completed and figures generated in the program GraphPad Prism. Tests for statistical significance were performed using unpaired two-tailed Student’s T test. p<0.05, **p<0.01, ***p<0.001, ns, not significant (p>0.05).

## Results

### BATF and BATF3 expression limit iTreg conversion

**Figure 1:**
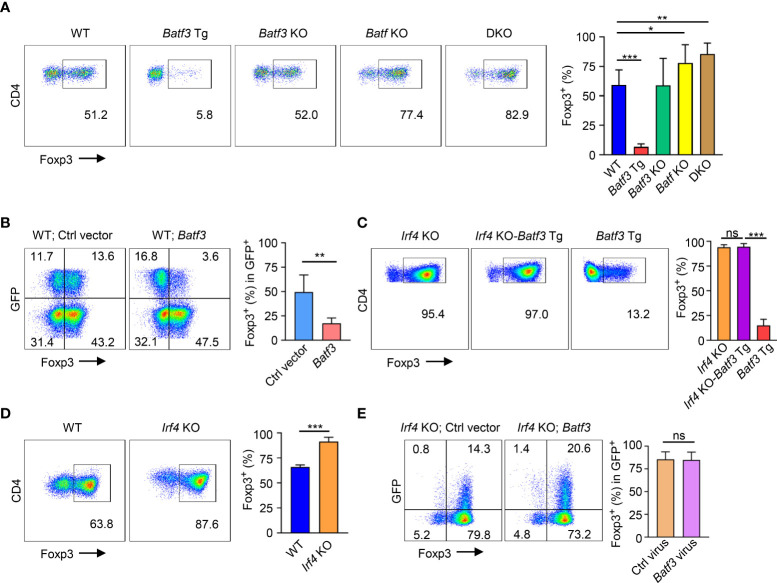
BATF3 and IRF4 on FOXP3 expression **(A, C, D)** Naïve CD4+ T cells were cultured for 72 hours in iTreg-inducing conditions. % FOXP3 shown as percentage of live CD4+ cells that are FOXP3+. n = 3. Data displayed as mean + S.D. **(B, E)** WT or *Irf4* KO naïve CD4+ T cells were activated overnight under T_0_ conditions and then transduced with either *Batf3*-flag or empty vector (Ctrl) GFP virus. Following transduction, cells were cultured in iTreg-inducing conditions for 48 hours. % FOXP3 shown as percentage of virus+ cells. n = 5 **(B)** or 3 **(E)**. Data displayed as mean + S.D. Differences in FOXP3 expression were determined using unpaired two-tailed Student’s T test. *p<0.05, **p<0.01, ***p<0.001, ns, not significant (p>0.05).

In CD4+ T cells cultured under iTreg-inducing conditions (i.e., TGF-β + IL-2), overexpression of BATF3 using a transgenic (*Batf3* Tg) model significantly inhibited FOXP3 expression (**Figure 1A**). Knockout of BATF3 did not significantly increase FOXP3 expression, but knockout of BATF or double knockout (DKO) of both BATF and BATF3 did increase FOXP3 expression. Although it did not reach statistical significance, DKO consistently demonstrated slightly increased FOXP3 expression compared to *Batf* KO (**Figure 1A**) indicating that although BATF is likely the dominant transcription factor expressed in our model, BATF and BATF3 can compensate for each other. Accordingly, retroviral overexpression of BATF3 or BATF inhibited FOXP3 expression (**Figure 1B**; **Supplementary Figure 1A**). We chose to primarily utilize overexpression of BATF3 due to the availability of our *Batf3* Tg model and use BATF3 to represent the *Batf*/*3* family of transcription factors.

### BATF3 is dependent on IRF4 to inhibit FOXP3

Because BATF3 is known to interact with IRF4, we sought to understand what role IRF4 might play in the inhibition of FOXP3 expression by BATF3. To do so, we crossed *Irf4* knockout (*Irf4* KO) and *Batf3* Tg mice to create *Irf4* KO-*Batf3* Tg mice. Overexpression of BATF3 without IRF4 failed to inhibit FOXP3 expression (**Figure 1C**). Similar to DKO cells, *Irf4* KO T cells were more readily induced to become FOXP3+ iTregs compared to WT cells (**Figure 1D**). In contrast to BATF3, retroviral overexpression of IRF4 did not significantly reduce FOXP3 expression (**Supplementary Figure 1B**). Retroviral overexpression of BATF3 in *Irf4* KO cells was also unable to inhibit FOXP3 expression (**Figure 1E**). These data indicate that both BATF3 and IRF4 control FOXP3 expression, and that BATF3 inhibits FOXP3 in an IRF4-dependent manner.

### BATF3 inhibition of FOXP3 is dependent on interactions with IRF4

**Figure 2:**
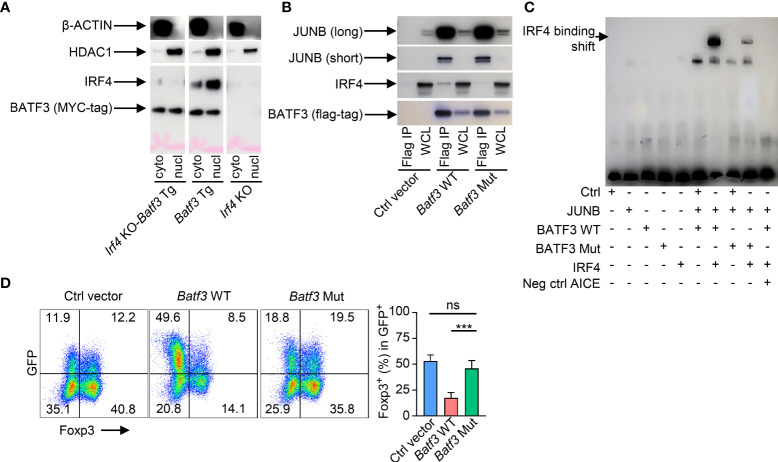
BATF3/IRF4 interactions are necessary for BATF3-mediated FOXP3 inhibition. **(A)** Naïve CD4+ T cells were activated and differentiated in iTreg-inducing conditions. After 48 hours, cells were collected and nuclear and cytoplasmic proteins extracted. Blot representative of 2 independent experiments. **(B)** WT naïve CD4+ T cells were activated overnight in To conditions and then transduced with empty vector (Ctrl), WT *Batf3*-flag (*Batf3* WT), or mutant *Batf3*-flag (*Batf3* Mut) virus. Cells were incubated for 48 hours in iTreg conditions before extracting proteins using IP lysis buffer. A sample of the whole cell lysate (WCL) was taken, and then the remaining samples were immunoprecipitated using anti-FLAG antibody. IP and WCL samples run on western blot stained with anti-FLAG, anti-IRF4, and anti-JUNB (short and long exposures). Blot representative of 2 independent experiments. **(C)** 293 T cells were transduced with viruses containing respective proteins or empty vector (Ctrl). Nuclear protein was extracted after 48 hrs. Biotin-labeled DNA containing the BATF/IRF4 AICE binding motif from the *ctla4* gene was incubated with the respective nuclear extracts and anti-IRF4 antibody before running in electromobility shift assay (EMSA). Neg ctrl AICE is non-biotin labeled and added 200:1 against biotin labeled. Blot representative of 3 independent experiments. **(D)** WT naïve CD4+ T cells were activated overnight in T_0_ conditions and then transduced with respective viruses. Cells were incubated for 48 hours in iTreg conditions. % FOXP3 shown as percentage of virus+ cells. n = 4. Data displayed as mean + S.D. Differences in FOXP3 expression were determined using unpaired two-tailed Student’s T test. ***p<0.001, ns, not significant (p>0.05).

As *Irf4* KO cells were more easily induced to FOXP3 expression, we sought to further understand if the IRF4-dependent manner of BATF3-mediated FOXP3 inhibition was due specifically to interactions of IRF4 with BATF3 or due to other cell-intrinsic factors within the *Irf4* KO cells. We confirmed that BATF3 is able to enter the nucleus in the absence of IRF4 (**Figure 2A**). IRF4 binds the DNA weakly on its own, and four specific residues on BATF have been shown to be critical to IRF4’s ability to bind AP-1/IRF composite elements (AICE) (25). To test the importance of BATF3/IRF4 interactions, we cloned mutations into the four analogous residues in BATF3 (H57Q, E58A, Q65D, E79K. *Batf3* Mut). CoIP demonstrated that the *Batf3* mutations did not inhibit BATF3’s ability to partner with JUNB but decreased its interactions with IRF4 (**Figure 2B**). EMSA assays confirmed that BATF3 and JUNB heterodimers are able to bind to the AICE sequence without IRF4 and allow the integration of IRF4 into these sequences. Similarly, BATF3 Mut/JUNB was able to bind the known AICE DNA sequence but decreased the ability of IRF4 to do so (**Figure 2C**). In contrast to BATF3 WT, BATF3 Mut did not significantly reduce FOXP3 expression (**Figure 2D**). Thus, BATF3/IRF4 interactions are critical to BATF3-mediated inhibition of FOXP3 expression.

### BATF3/IRF4 interactions alter global IRF4 binding, including in the FOXP3 super enhancer

**Figure 3:**
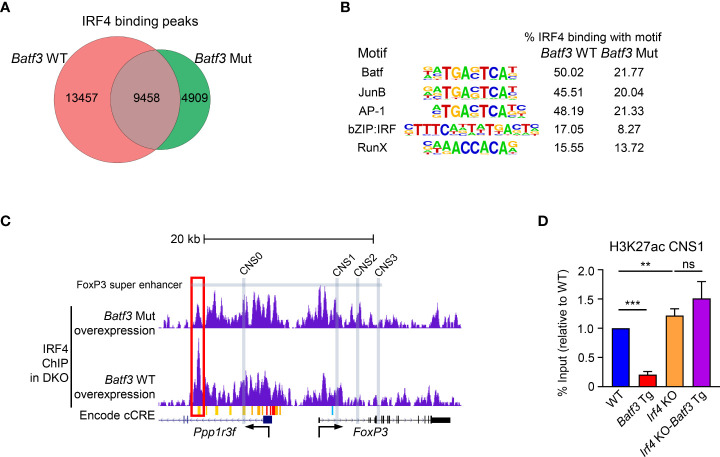
IRF4 ChIP-seq and H3K27ac ChIP-qPCR. **(A–C)**: Naïve DKO CD4+ T cells were activated overnight in T_0_ conditions before transduction with *Batf3* WT or *Batf3* Mut viruses. Transduced cells were cultured in iTreg media for 24 hrs before collection, sorting for virus + cells, and then fixing for ChIP-seq of IRF4. **(A)** Number of unique IRF4 binding peaks in cells transduced with *Batf3* WT or *Batf3* Mut. **(B)** IRF4 binding motifs in *Batf3* WT and *Batf3* Mut transduced cells. **(C)** IRF4 binding in the *Foxp3* super enhancer and locus. Differential IRF4 binding peak marked with red box. Super enhancer as defined by Kitagawa et al. Encode cCREs as displayed in UCSC browser. CNS0-3 marked with gray boxes. **(D)** Naïve CD4+ T cells were activated and differentiated in iTreg inducing media for 48 hrs before ChIP-qPCR for H3K27ac of CNS1 of the *Foxp3* locus. Data expressed as mean and S.D. of % input normalized to WT % input of 4 independent experiments. Statistical significance calculated using unpaired two-tailed Student’s T test. **p<0.01, ***p<0.001, ns, not significant (p>0.05).

Taken together, these data support the hypothesis that elevated expression of BATF3 recruits IRF4 to key DNA sequences that inhibit FOXP3 expression. To test this hypothesis, we overexpressed BATF3 WT and BATF3 Mut in DKO CD4+ T cells and performed ChIP-seq for IRF4. The use of DKO T cells ensures that endogenous BATF or BATF3 do not alter the IRF4 binding profile. We observed that the total number of IRF4 binding peaks was reduced by BATF3 Mut, with a majority of IRF4 binding in the BATF3 Mut sample shared with BATF3 WT whereas the majority of IRF4 binding in the BATF3 WT was unique (**Figure 3A**). We utilized a homer motif analysis to confirm that IRF4 bound primarily at AP-1/BATF binding sites. The top motifs of IRF4 binding in both BATF3 WT and BATF3 Mut conditions were variations of the AP-1/BATF/JUNB binding motif. However, we found that the percentage of target sequences with the motif was dramatically decreased with BATF3 Mut overexpression compared to BATF3 WT, consistent with BATF3 being a necessary binding partner for IRF4 in T cells. In contrast, the top non-AP-1 motif that IRF4 bound, RUNX, was approximately equally represented in both BATF3 WT and BATF3 Mut samples (**Figure 3B**).

No IRF4 binding peaks were found within the *Foxp3* gene body or the known *Foxp3* enhancers CNS0, CNS1, CNS2, or CNS3. However, IRF4 did bind a cis-regulatory elements (cCREs), identified by the ENCODE consortium as E0923691, located approximately 14kb upstream of the *Foxp3* TSS in a BATF3-interaction dependent manner (**Figure 3C**). This locus is found within the *Foxp3* super enhancer previously identified by Kitagawa et al. (7) As CNS1 has been identified as the critical enhancer for TGF-β mediated FOXP3 expression, we performed ChIP-qPCR for H3K27ac of the CNS1 region to determine how BATF3 and IRF4 binding within the *Foxp3* super enhancer alter activation of this enhancer. Concordant with our FOXP3 expression results, overexpression of BATF3 significantly decreased H3K27ac of CNS1 in an IRF4-dependent manner (**Figure 3D**). Thus, BATF3 overexpression recruits IRF4 to the *Foxp3* super enhancer and prevents activation of CNS1 to prevent FOXP3 expression.

### BATF3 and IRF4 interactions are necessary for glycolytic remodeling of activated T cells

**Figure 4:**
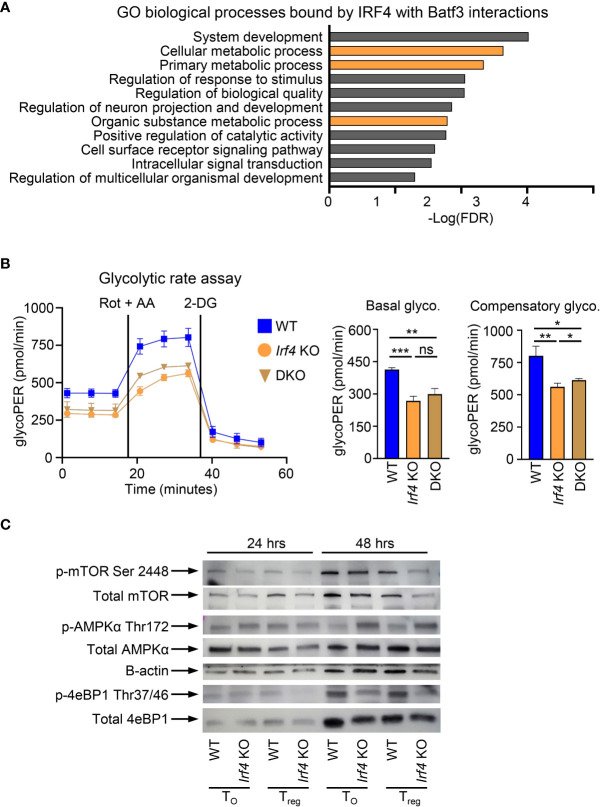
BATF3/IRF4 interactions necessary for maintenance of cellular metabolism upon T cell activation. **(A)** Gene ontology analysis of pathways enriched by BATF3/IRF4 interactions generated from list of genes bound by IRF4 only in *Batf3* WT sample. Data presents all upregulate pathways with –Log(FDR) < 0.05. FDR calculated by Benjamini-Hochberg False Discovery Rate. Metabolic pathways highlighted in orange. **(B)** Glycolytic rate for CD4+ T cells activated and cultured for 48 hrs in T_0_ conditions. GlycoPER represents proton efflux rate due to glycolysis. Data represents mean and S.D. of 3 independent experiments, each consisting of 4-6 technical replicates averaged together. Statistical significance calculated using unpaired two-tailed Student’s T test for the three averages of the independent experiments. *p<0.05, **p<0.01, ***p<0.001, ns, not significant (p>0.05). **(C)** Western Blot of WT or *Irf4* KO CD4+ T cells activated and cultured for 24 or 48 hrs in iTreg or T_0_ conditions and stained for respective phosphorylated or total proteins. Representative of 2 independent experiments.

In addition to binding within the *Foxp3* super enhancer, we sought to determine if additional cellular processes under the control of BATF3 and IRF4 interactions contribute to FOXP3 inhibition. To assess this, we compiled the list of genes from our ChIP-seq data which were only bound by IRF4 with BATF3 WT. This list of 2,371 genes was analyzed by gene ontology analysis for biological processes (37, 38). Significantly enriched processes were determined as p<0.05 using the Benjamini-Hochberg procedure correction for False Discovery Rate in multiple analyses. Among the biological processes most significantly enriched in IRF4 binding by BATF3/IRF4 interactions were cellular metabolic processes (FDR = 0.00231), implying that IRF4 binding is critical to cellular metabolic reprograming (**Figure 4A**). Thus, we hypothesized that metabolic upregulation in response to TCR signaling may represent an additional axis through which BATF3/IRF4 interactions control FOXP3 expression and stability.

Glycolysis is dramatically upregulated following T cell activation and is inhibitory toward FOXP3 expression. To determine the impact of BATF3 and IRF4 on glycolysis, we performed a glycolytic rate assay on WT, DKO, and *Irf4* KO cells. *Irf4* KO and DKO T cells showed a significant decrease in glycolytic activity at baseline and in the absence of the electron transport chain, demonstrating a critical role for these transcription factors in glycolytic upregulation upon T cell activation (**Figure 4B**). We confirmed the importance of IRF4 to T cell metabolism by examining phosphorylation of metabolic mediators in WT and *Irf4* KO cells cultured under T_0_ or iTreg conditions for 24 or 48 hrs. The decrease in glycolytic rate is accompanied by a decrease in mTOR activity, as measured in 4E-BP1 phosphorylation, and an increase in AMPKα phosphorylation (**Figure 4C**). These alterations in phosphorylation were seen whether *Irf4* KO cells were cultured in T_0_ or iTreg conditions, demonstrating an inherent defect in cellular energy levels without IRF4 that is independent of iTreg conversion. These differences in metabolic states were observed at 48 hrs, but not 24 hrs, indicating that IRF4 is critical to sustained metabolic remodeling in response to TCR signaling (**Figure 4C**).

### BATF3/IRF4 mediated glycolytic reprograming prevents stable FOXP3 expression

**Figure 5:**
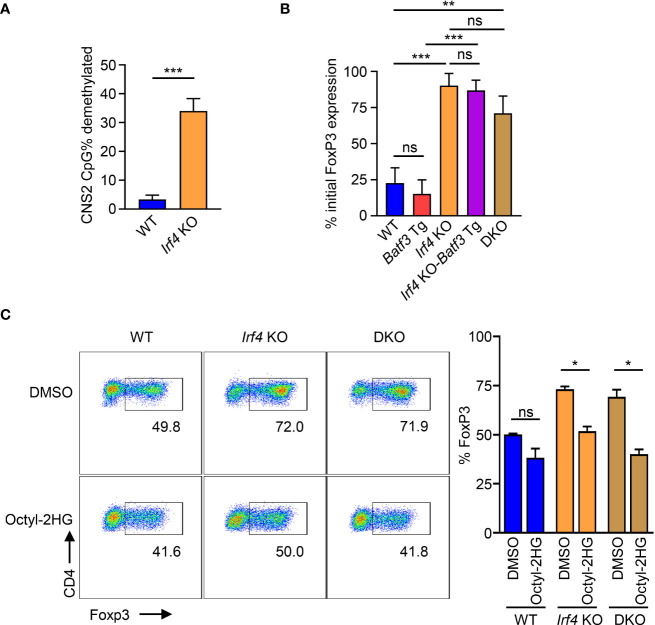
Control of FOXP3 expression through CNS2 methylation. **(A)** % CpG demethylation of the CNS2 *Foxp3* enhancer. *Irf4* KO and WT CD4+ T cells were activated and differentiated for 72 hrs in iTreg media before bisulfite sequencing of the CNS2 region. Data expressed as mean + S.D. of 3 independent experiments. **(B)** FOXP3 stability: Naïve CD4+ T cells were activated and differentiated for 72 hrs in iTreg media before being reactivated by APCs and recultured for 72 hrs in T_0_ media. Data expressed as mean and S.D. of % of FOXP3 expression before reactivation by APC. n=3. (**(C)**) FOXP3 expression of cells activated and differentiated for 72 hrs in iTreg media in the presence of 0.5 mM Octyl-2HG or DMSO vehicle control. Data expressed as mean + S.D of 2 independent experiments. Statistical significance determined by unpaired two-tailed Student’s T test. *p<0.05, **p<0.01, ***p<0.001, ns, not significant (p>0.05).

AMPK has been shown to phosphorylate and stabilize TET2, which is necessary for demethylation of CNS2 and stable FOXP3 expression (39). Our findings that IRF4 contributes to the sustaining of glycolytic metabolism with the resulting phosphorylation of AMPKα suggest that the metabolic reprogramming under IRF4 and BATF3 may contribute to FOXP3 stability. Bisulfite sequencing of WT and *Irf4* KO iTregs confirmed that *Irf4* KO T cells have greater demethylation of CNS2 (**Figure 5A**). We tested the stability of FOXP3 expression by differentiating iTregs for 72 hrs and then restimulating with APCs in T_0_ conditions. We found that *Irf4* KO and DKO iTregs maintained greater FOXP3 expression (**Figure 5B**), consistent with increased demethylation of CNS2. Addition of the TET enzyme inhibitor 2-hydroxyglutarate significantly decreased FOXP3 expression in *Irf4* KO and DKO cells, demonstrating the importance of TET enzymes to the expression and stability of FOXP3 expression in *Irf4* KO and DKO cells (**Figure 5C**). Thus, IRF4 and BATF3 constrain FOXP3 expression and stability in recently activated CD4+ T cells through the maintenance of glycolytic reprogramming which in turn limits AMPKα phosphorylation and TET enzyme stabilization.

## Discussion

Activation of CD4+ T cells, and their polarization toward an inflammatory or regulatory phenotype, is a critical axis of immune regulation. Herein, we investigated to role of IRF4 and BATF3, two complementary transcription factors known to be upregulated in response to TCR and CD28 signaling. Inhibition of FOXP3 is positively correlated with the expression of BATF or BATF3. This inhibition is dependent on direct interactions of BATF transcription factors with IRF4. In contrast, high IRF4 expression without a corresponding increase in BATF/3 did not significantly reduce FOXP3 expression, but its absence allowed potent conversion of cells to iTregs, similar to DKO of BATF and BATF3. These data indicate that inhibition of FOXP3 is primarily determined by expression of BATF/3, which likely outcompete FOS for JUN binding and allow integration of IRF4 into AP-1 sequences that inhibit FOXP3 expression. IRF4 has been identified as a rheostat for strength of TCR signaling, and BATF is reported to be upregulated in response to CD28 signaling and other cytokine cues but downregulated in response to TGF-β (15, 19, 23). Thus, BATF/3 levels resulting from costimulation through CD28, particularly in the absence of TGF- β, may be critical to determining FOXP3 expression in activated CD4+ cells, and consequently the Treg phenotype, consistent with reports that TCR signaling is conducive to FOXP3 expression in the absence of CD28 signaling and that anergic cells, which experience TCR signaling in the absence of CD28, may be Treg precursors (3, 40).

We found IRF4 binds to a regulatory region upstream within the *Foxp3* super enhancer in a BATF3 dependent manner. BATF3-dependent binding of IRF4 at this region coincides with decreased H3K27ac acetylation of the TGF-β sensitive enhancer CNS1, which also lies within the *Foxp3* super enhancer. BATF3-mediated closure of CNS1 dramatically decreases conversion to FOXP3+ cells despite the presence TGF-β and IL-2. Thus, this regulatory element represents a potential silencer region that imbues TCR/CD28-signaling-dependent inhibition of FOXP3 through BATF/IRF4 transcription factors. Super enhancer regulation is still poorly understood, but hierarchical control wherein pioneer enhancer regions allow activation of subsequent regions and super enhancer formation has been demonstrated, including in the *Foxp3* locus (9). Accordingly, BATF3 and IRF4 binding within the super enhancer may represent a mechanism of hierarchical control of the super enhancer, although additional studies will be needed to confirm the importance of this locus to the *Foxp3* super enhancer formation and silencing.

We additionally found that BATF family and IRF4 interactions are necessary to sustaining the glycolytic metabolic remodeling that occurs upon T cell activation, with severe decreases in both basal and maximum glycolysis and an increase in AMPKα phosphorylation observed in the absence of these transcription factors. Changes in phosphorylation of AMPKα and 4E-BP1 were present at 48 hrs but not at 24 hrs, consistent with the expression of BATF/IRF4 upon, not before, T cell activation. AMPKα acts as an inhibitor of mTOR and promotes fatty acid oxidation and TET stabilization – conditions which are favorable to FOXP3 expression and demethylation of CNS2 (41, 42). Several papers have previously demonstrated the importance of this demethylation to the Th17/iTreg axis (43, 44), and BATF and IRF4 are known to promote the Th17 phenotype. Taken together, these data provide a metabolic mechanism whereby IRF4 and BATF transcription family members translate activating signals from the TCR and CD28 into metabolic commitment of Teff functions and inhibition of FOXP3. The lack of this commitment allows for demethylation of CNS2 and commitment to a regulatory phenotype.

Our studies highlight the importance of interactions between BATF transcription factors and IRF4 in T cell fate decisions after activation, particularly inhibition of FOXP3 and commitment to an effector T cell lineage. They do this both by directly interacting at the *Foxp3* locus and by upregulating cellular metabolic pathways. Our studies have focused primarily on the role of these transcription factors in iTregs. Additional studies will be needed to understand their roles in both peripheral and thymic Tregs *in vivo*, as well as how balancing the expression of these transcription factors upon T cell activation integrates with other signals and cellular processes to allow functional iTreg development. Such an understanding may yield important insights into how activated T cells are driven toward inflammatory or regulatory phenotypes and the resultant regulation of the immune system.

## Data availability statement

The datasets presented in this study have been deposited in NCBI's Gene Expression Omnibus and are accessible through GEO Series accession number GSE211182 (https://www.ncbi.nlm.nih.gov/geo/query/acc.cgi?acc=GSE211182).

## Ethics statement

The animal study was reviewed and approved by Houston Methodist Animal Care Committee.

## Author contributions

PA oversaw, designed, and conducted the experiments and wrote the manuscript. MW, LZ, and YY assisted in conducting various experiments. XX assisted in designing, completion, and interpretation of many of the experiments. XC assisted in the design of CoIP and EMSA experiments. GW assisted in vector and plasmid generation. XZ conducted many experiments necessary for hypothesis generation and helped construct plasmids and primers. AZ and DH assisted in the design, completion, and interpretation of metabolic experiments. WC provided valuable discussion in interpreting results and designing figures. XL oversaw the experiments, interpreted results, and helped edit figures and write the manuscript. All authors contributed to the article and approved the submitted version.

## Funding

This work was supported by the NIH grants R01AI106200 and R01AI129906.

## Acknowledgments

We acknowledge the flow cytometry core at Houston Methodist Research Institute for excellent service and Laurie Minze for operational support. We thank the many members of the Houston Methodist Immunobiology and Transplant Center for insightful discussion.

## Conflict of interest

The authors declare that the research was conducted in the absence of any commercial or financial relationships that could be construed as a potential conflict of interest.

## Publisher’s note

All claims expressed in this article are solely those of the authors and do not necessarily represent those of their affiliated organizations, or those of the publisher, the editors and the reviewers. Any product that may be evaluated in this article, or claim that may be made by its manufacturer, is not guaranteed or endorsed by the publisher.
