# Supplementary material for: Suppression of FOXP3 expression by the AP-1 family transcription factor BATF3 requires partnering with IRF4
Source: Front Immunol. 2022 Aug 25;13:966364. doi: 10.3389/fimmu.2022.966364 (PMC9452699; doi:10.3389/fimmu.2022.966364)
Supplement: Supplementary file 1 [file DataSheet_1.docx]

**Supplementary Tables**

**Supplementary Table 1:** Primers and DNA sequences

| Region | Forward | Reverse |
| --- | --- | --- |
| CNS1 | TGAGCACCTACCATCATT | CCTCTCTGTACCTCCATTT |
| Batf3 H57Q E58A | ACAAGCTCCAGGCGGAGCACGAG | CAGCCTTCTGGGTCTGCT |
| Batf3 Q65D | GAGAGCCTGGAGGACGAGAACTCTGT | GTGCTCCTCGTGGAGCTT |
| Batf3 E79K | GAAGCTGAAGAAGGAGCTGCG | GAAATCTCCCTGCGCAGC |
| CTLA4 AICE Duplex | /5BiosG/TCACGTACAGTATTAGTCAT CCCGAAACCTCTAAGGCAAG | /5BiosG/CTTGCCTTAGAGGTTTCGGGA TGACTAATACTGTACGTGA |
| Neg control AICE duplex | TCACGTACAGTATTAGTCAT CCCGAAACCTCTAAGGCAAG | CTTGCCTTAGAGGTTTCGGGA TGACTAATACTGTACGTGA |

**Supplementary Table 2:** Antibodies

| Target | Source | Identifier |
| --- | --- | --- |
| **Western Blot/CoIP** |  |  |
| Β-actin | Santa Cruz | sc-4778 (C4) |
| HDAC1 | Santa Cruz | sc-81598 (10E2) |
| H3 | Santa Cruz | sc-517576 (1G1) |
| IRF4 | CST | D9P5H |
| MYC-Tag | CST | 9B11 |
| JunB | CST | C37F9 |
| p-mTOR (Ser2448) | CST | D9C2 |
| mTOR | CST | 7C10 |
| p-AMPKα (Thr172) | CST | 40H9 |
| AMPKα | CST | D5A2 |
| p-4E-BP1 (Thr37/46) | CST | 53H11 |
| 4E-BP1 | CST | 53H11 |
| Flag | Sigma | M2 |
| **Flow Cytometry** |  |  |
| Flag-APC | BioLegend | L5 |
| FoxP3-PE | Invitrogen | FJK-16s |
| FoxP3-eFluor450 | Invitrogen | FJK-16s |
| CD4-PECy7 | Invitrogen | GK1.5 |
| Zombie Aqua | Invitrogen |  |
| CD45.1-APC | Invitrogen | A20 |
| CD45.2-APCCy7 | Invitrogen | 104 |
| GFP-PE | Invitrogen | FM264G |
| CD25-PE | Invitrogen | PC61 |
| **ChIP** |  |  |
| IRF4 | CST | D9P5H |
| H3K27ac | Active Motif | 39133 |
