# Supplementary material for: Suppression of FOXP3 expression by the AP-1 family transcription factor BATF3 requires partnering with IRF4
Source: Front Immunol. 2022 Aug 25;13:966364. doi: 10.3389/fimmu.2022.966364 (PMC9452699; doi:10.3389/fimmu.2022.966364)
Supplement: Supplementary file 2 [file Image_1.pdf]

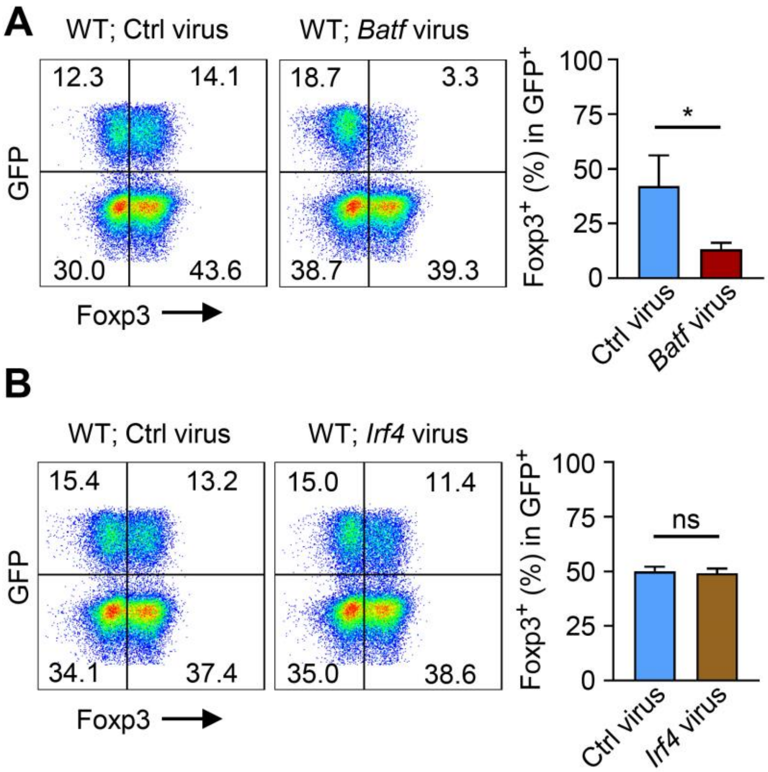

Supplemental Figure 1 (with Figure 1): BATF and IRF4 overexpression on FOXP3 expression

(A, B): WT naïve CD4+ T cells were activated overnight under T_0 conditions and then transduced with either *Batf*-flag (A), *Irf4* (B) or empty vector (Ctrl) GFP virus. Following transduction, cells were cultured in iTreg-inducing conditions for 48 hours. % FOXP3 shown as percentage of virus+ cells. n = 3 (A) or 5 (B). Data displayed as mean + S.D. Differences in FOXP3 expression were determined using unpaired two-tailed Student's T test. * = $p<0.05$, ns = not significant ($p>0.05$).
